# Deep learning-based automated diagnosis of temporomandibular joint anterior disc displacement and its clinical application

**DOI:** 10.3389/fphys.2024.1445258

**Published:** 2024-12-13

**Authors:** Yue Yu, Shu Jun Wu, Yao Min Zhu

**Affiliations:** Department of Oral & Maxillofacial Surgery, Shenzhen Stomatology Hospital, Affiliated to Shenzhen University, Shenzhen, Guangdong Province, China

**Keywords:** temporomandibular joint, anterior disc displacement, magnetic resonance imaging, deep learning, automated diagnosis

## Abstract

**Introduction:**

This study aimed to develop a deep learning-based method for interpreting magnetic resonance imaging (MRI) scans of temporomandibular joint (TMJ) anterior disc displacement (ADD) and to formulate an automated diagnostic system for clinical practice.

**Methods:**

The deep learning models were utilized to identify regions of interest (ROI), segment TMJ structures including the articular disc, condyle, glenoid fossa, and articular tubercle, and classify TMJ ADD. The models employed Grad-CAM heatmaps and segmentation annotation diagrams for visual diagnostic predictions and were deployed for clinical application. We constructed four deep-learning models based on the ResNet101_vd framework utilizing an MRI dataset of 618 TMJ cases collected from two hospitals (Hospitals SS and SG) and a dataset of 840 TMJ MRI scans from October 2022 to July 2023. The training and validation datasets included 700 images from Hospital SS, which were used to develop the models. Model performance was assessed using 140 images from Hospital SS (internal validity test) and 140 images from Hospital SG (external validity test). The first model identified the ROI, the second automated the segmentation of anatomical components, and the third and fourth models performed classification tasks based on segmentation and non-segmentation approaches. MRI images were classified into four categories: normal (closed mouth), ADD (closed mouth), normal (open mouth), and ADD (open mouth). Combined findings from open and closed-mouth positions provided conclusive diagnoses. Data augmentation techniques were used to prevent overfitting and enhance model robustness. The models were assessed using performance metrics such as precision, recall, mean average precision (mAP), F1-score, Matthews Correlation Coefficient (MCC), and confusion matrix analysis.

**Results:**

Despite lower performance with Hospital SG’s data than Hospital SS’s, both achieved satisfactory results. Classification models demonstrated high precision rates above 92%, with the segmentation-based model outperforming the non-segmentation model in overall and category-specific metrics.

**Discussion:**

In summary, our deep learning models exhibited high accuracy in detecting TMJ ADD and provided interpretable, visualized predictive results. These models can be integrated with clinical examinations to enhance diagnostic precision.

## 1 Introduction

Temporomandibular disorder (TMD) comprises various conditions that result in pain and functional impairment of the masticatory muscle and the temporomandibular joint (TMJ) (Ikai et al., 1997). Epidemiological studies indicate that TMDs affect a significant portion of the adult population. Estimates of TMD prevalence in adults generally range from 5% to 12% globally. Anterior Disc Displacement (ADD) is the most common subtype of TMD, with an estimated occurrence of 30%–60% among TMD patients (Manfredini, 2009; Hugoson and Magnusson, 2011; Pantoja et al., 2019; Valesan et al., 2021). Normally, the articular disc should remain positioned above the condyle in open and closed mouth positions. ADD is diagnosed when the disc displaces anteriorly in either position (Ikeda and Kawamura, 2013; Freudenthaler et al., 2022). The articular disc, situated between the condyle and the glenoid fossa, is highly resilient and stable, helping to alleviate the pressure exerted on the TMJ during mandibular movements such as mastication. Consequently, positional or morphological alterations of the disc can lead to the onset and progression of TMD (Hall et al., 1995; Kurita et al., 2001; Takaoka et al., 2021). Studies suggest that TMJ ADD can lead to malocclusion, mandibular retrusion, and facial asymmetry, potentially leading to psychosocial issues for patients (Bósio et al., 1998; Nebbe et al., 1999a; Nebbe et al., 1999b). Therefore, this study primarily focuses on the TMJ ADD.

Magnetic resonance imaging (MRI) is widely recognized as the criterion standard diagnostic modality for diagnosing TMJ disc displacement, as it provides detailed visualization of the anatomical structures and pathological changes of the TMJ (Honda et al., 2001; Schiffman et al., 2014). However, the complexity of TMJ MR image analysis is increased due to the often unclear and low-contrast depiction of TMJ components and surrounding tissues, as well as the morphological changes of the disc caused by disease progression. MRI evaluation is typically subjective, with diagnoses varying depending on the examiner’s experience and the MR sequences used. Therefore, standardized MRI analysis is crucial to ensure diagnostic accuracy and reproducibility.

Deep learning methods utilizing Convolutional Neural Networks (CNNs) represent the forefront of artificial intelligence technology. Due to their superior performance in medical image recognition tasks, encompassing both segmentation and classification, and the extensive accessibility of open-source frameworks, CNNs have become a predominant technology within the domain of medical image analysis (Lee et al., 2022; Shafiq and Gu, 2022). The development of effective predictive models for automatically detecting specific pathological features in MRI requires training CNN algorithms on large datasets of annotated images.

A literature review suggests that CNN-based models have become pivotal in segmenting craniofacial bone structures from MR images, with U-Net++ and U-Net variants particularly noted for their proficiency in capturing intricate anatomical details (Nie et al., 2017; Li et al., 2022). Subsequent research has effectively utilized a range of deep learning approaches, including CNNs, U-Nets, U-Net++, U-Net3+, DeepLabV3+, and Segnet, for automating the segmentation of the TMJ disc in MR images (Ito et al., 2022; Nozawa et al., 2022; Min et al., 2024). Among these, while Segnet demonstrated comparative efficacy, DeepLabV3+ emerged as the most effective for advanced semantic segmentation tasks. Moreover, studies have employed deep learning and CNN-based methods for the automated classification and detection of anterior disc displacement (ADD) (Lee et al., 2022; Lin et al., 2022; Yoon et al., 2023). These classification models, predicated on VGG16 and Resnet architectures, have showcased promise in accurately identifying ADD. Additionally, an algorithm has been developed in research to predict TMJ disc perforation from MRI findings (Kim et al., 2021). This approach involves experienced observers interpreting MRI images to extract features related to disc morphology, bone marrow signal, the relationship between the disc and condyle, joint space, and changes in the condyle and fossa. These features are then utilized to construct and validate predictive models using random forest and Multilayer Perceptron (MLP) techniques. Despite its utility, manual feature extraction may be more suitable for tasks with well-defined features and smaller datasets. However, in our study, there is a need for models that can autonomously learn features from raw images to excel in complex tasks.

Considering our study’s dual objectives of segmenting and classifying TMJ MR images, primarily focusing on classification, we have selected Resnet101_vd as the backbone network due to its robust feature extraction capabilities. Resnet’s residual connections facilitate the training of deeply layered networks, enhancing feature extraction for complex medical imaging data. Resnet101’s faster training relative to VGG16, coupled with its superior performance on standard datasets like ImageNet in terms of accuracy and generalization, makes it an attractive choice. Moreover, its flexibility supports both classification and segmentation tasks, feasibly integrating with other models for feature sharing (He et al., 2016). For the segmentation model, considering the complex nature of medical images, especially MRIs, with their rich structural details and noise, we employ ResNet101_vd combined with DeepLabV3+. DeepLabV3+ utilizes atrous convolution and ASPP strategies to capture multi-scale features, which is essential for handling complex segmentation tasks and capturing fine details in medical images (Chen et al., 2018; Wang et al., 2024). Consequently, this study utilizes ResNet-101 as the backbone network, with the segmentation model being DeepLabV3 + ResNet-101, and employs ResNet101_vd for subsequent classification tasks.

**FIGURE 1:**
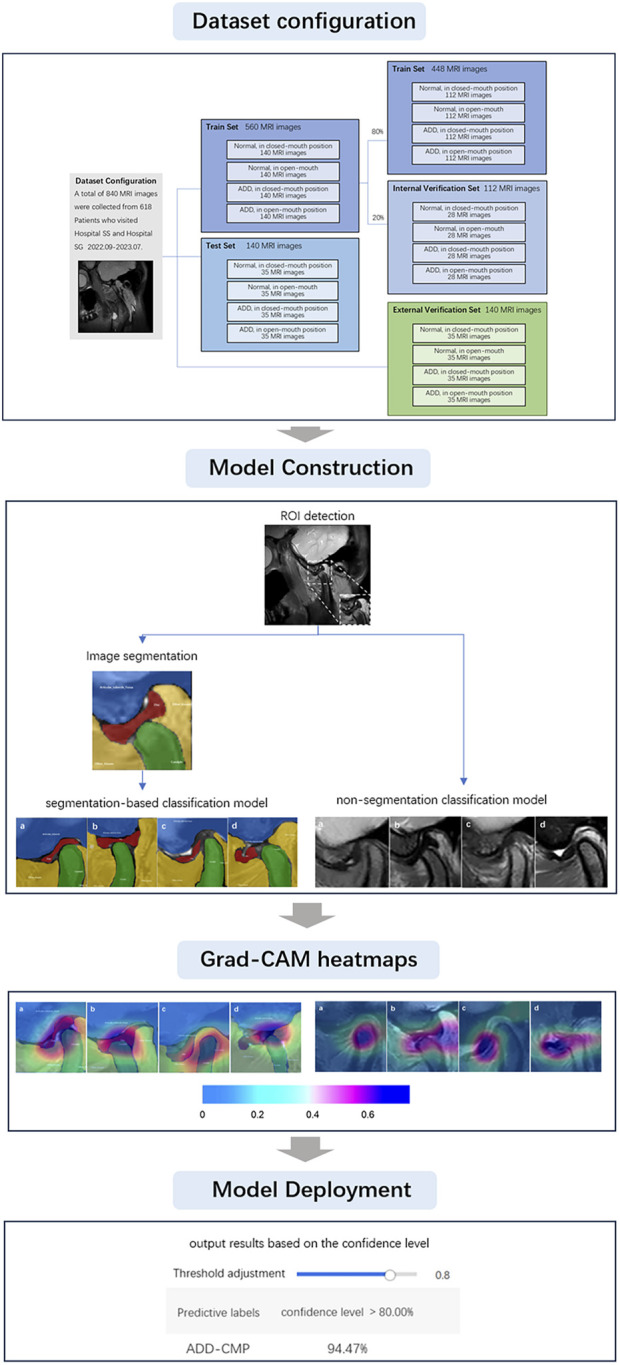
Diagrammatic representation of the research methodology.

Previous related studies have primarily focused on either image segmentation or image classification. Currently, there are no studies combining three key deep learning methods: Region of Interest (ROI) identification, image segmentation, and image classification, into a three-stage multi-task self-supervised learning approach to enhance diagnostic accuracy. Additionally, prior research has been conducted in laboratory settings, which may not fully reflect the benefits of clinical practice. In the clinical diagnosis of TMJ ADD, the real-time and accurate identification of the interaction between the mandibular condyle and the articular disc on MRI, and the prompt convey of this information to oral clinicians, is crucial for accurate diagnostic decisions and appropriate treatment. Therefore, the purpose of this study is to devise a deep learning-driven TMJ ADD MR image analysis method and to investigate the clinical applicability of deep learning networks. We have designed our system around four core deep learning models: an ROI detection model, a TMJ segmentation model, a segmentation-based classification model, and a non-segmentation classification model, to diagnose TMJ ADD. These models were deployed on high-performance computing hardware and are capable of generating artificial intelligence heatmaps, which serve as reliable diagnostic support for clinicians. The sequence of steps undertaken throughout the research is illustrated in Figure 1.

## 2 Materials and methods

### 2.1 Database establishment

This study was conducted based on clinical data from Shenzhen Stomatology Hospital (Hospital SS) and Shenzhen University General Hospital (Hospital SG) with approval from the Ethics Committee of Shenzhen Stomatology Hospital (Protocol Code: SZSK-20231221-1) and Shenzhen University General Hospital (Protocol Code: KYLL-20221217A). Written informed consent was obtained from all subjects involved in the study.

The study included TMJ MRI data from 618 patients, aged 19–40 years (mean age 28.9 ± 6.4 years), These patients presented with TMJ-related symptoms between September 2022 and July 2023. Professionals with more than a decade of expertise in TMDs utilized the Diagnostic Criteria for Temporomandibular Disorders (DC/TMD) to diagnose TMJ ADD cases. The exclusion criteria were as follows: 1. History of TMJ surgery; 2. History of tumors or facial fractures; 3. Systemic diseases that may affect TMJ function, such as rheumatic diseases and psychiatric disorders; 4. Lateral or medial disc displacement; 5. MRI results showing indeterminate signal intensity and contour of the joint disc structure; 6. Inconsistent diagnoses by different clinicians. For patients with multiple MRI scans, only the initial scan was included. These exclusion criteria were implemented to guarantee the precision and dependability of the study by minimizing potential confounding factors. A total of 840 TMJ MR images were included, and 700 and 140 images were obtained at the Hospital SS and Hospital SG, respectively. Images were divided into four groups: normal closed-mouth position (N-CMP), normal open-mouth position (N-OMP), anterior disc displacement closed-mouth position (ADD-CMP), and anterior disc displacement open-mouth position (ADD-OMP). Of these images, images obtained from Hospital SS were used with 140 images per group for the training dataset and 35 images per group for the test dataset. During CNN model training, the data was divided into a training set, which contained 80% of the data, and an internal validation set, which encompassed the remaining 20%, randomly for each experiment. All 140 images from Hospital B were used for external validation.

### 2.2 Magnetic resonance imaging examination

Since the TMJ disc is most distinctly visualized on proton density-weighted imaging (PDWI) (Gibbs and Simmons, 1998), this study exclusively utilized PDWI. For this study, MR images of patients at Hospital SS were obtained using a 1.5 T scanner (uMR; United Imaging, Shanghai, China) equipped with a TMJ coil. Imaging parameters were as follows: PDWI was conducted with a repetition time (TR) of 2,300 ms and an echo time (TE) of 47 ms; matrix size 312 × 312; field of view (FOV) 282 × 120 mm; slice thickness of 2.5 mm; and slice gap of 0.1 mm. In Hospital SG, images were obtained with a 3T MRI scanner (Magnetom Skyra, Siemens Healthineers, Erlangen, Germany) equipped with a head and neck coil. Imaging parameters were as follows: PDWI was conducted with a TR of 2,200 ms and a TE of 78 ms; matrix size 320 × 192; FOV 120 × 120 mm; slice thickness of 2.0 mm; and slice gap of 0.2 mm.

The focus of our investigation was to enhance diagnostic accuracy of TMJ ADD, hence the primary emphasis was placed on the oblique sagittal MRI positioning images. Sagittal scans were oriented parallel to the anteroposterior direction of the mandibular condyle head. MRI was conducted at maximum mouth open position and closed-mouth (intercuspal) position. Odd-numbered slices were acquired to ensure the midline image was central, with a minimum of 7 images obtained on each side (Johnson et al., 2022; Fu et al., 2020). For each patient, the image closest to the long axis and transverse axis of the condyle, with the condyle located within the central one-third of the image, was manually selected.

### 2.3 Data description and preprocessing

**FIGURE 2:**
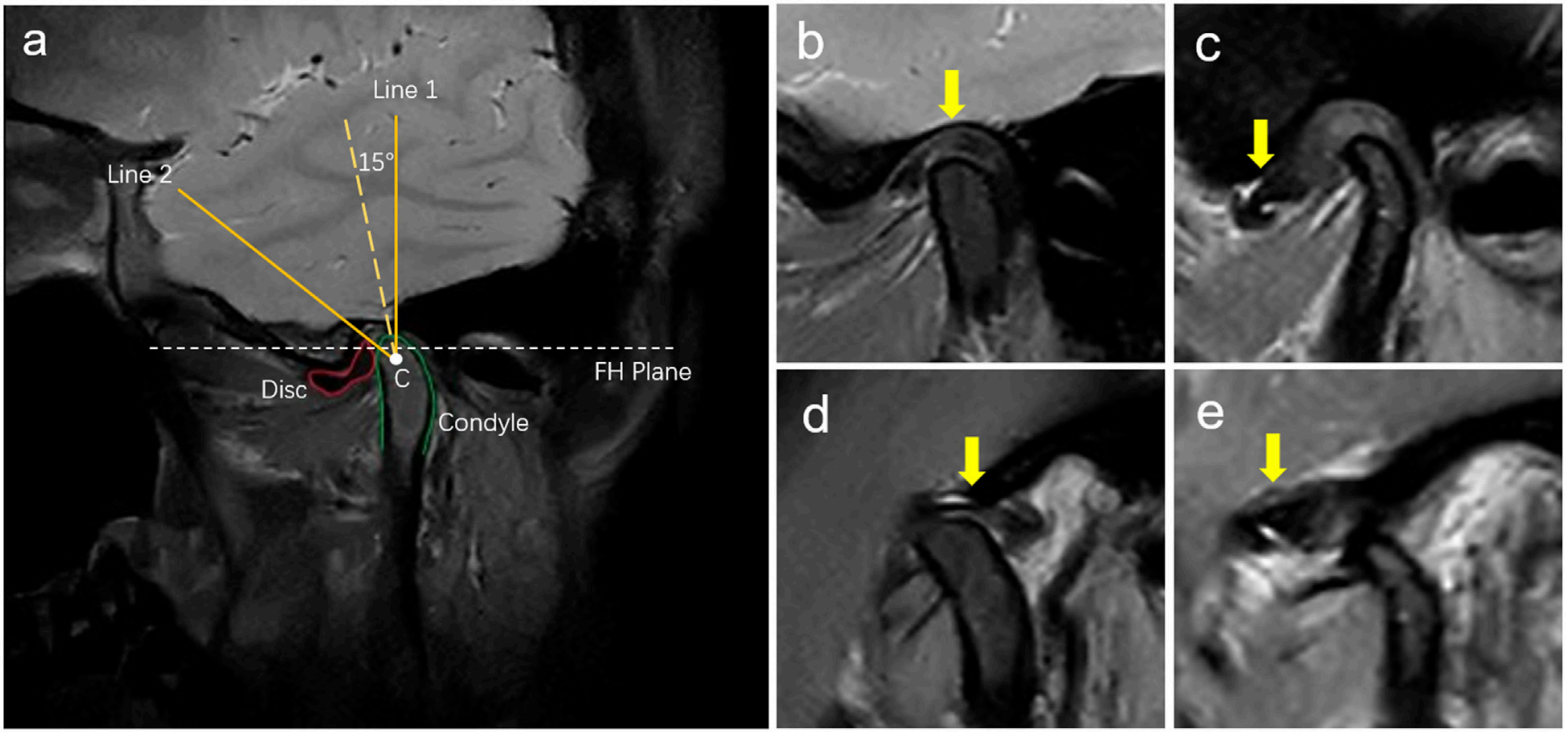
Diagnosis of disc displacements in MRIs. **(A)** MRI diagnostic method for TMJ ADD. C is the center of the condylar head; Line 1 is the line passing over the condylar apex and point C; Line 2 is the line passing over the posterior margin of the posterior band of the TMJ disc and point C; **(B)** N-CMP; **(C)** ADD-CMP; **(D)** N-OMP; **(E)** ADD-OMP. The arrow points to the location of the TMJ disc.

MRI diagnostic criteria for disc displacement: At the closed-mouth position, a line connecting the apex of the condyle and the center of the condylar head defines line 1, while a line connecting the posterior edge of the disc to the center of the condylar head defines line 2. ADD is defined when the angle between these two lines exceeds 15°. At the maximum mouth open position, the disc’s intermediate zone should be situated between the condylar head and the articular tubercle (Schiffman et al., 2014; Qian et al., 2023). If the posterior band of the disc was anterior to the condylar head, it was considered ADD (Figure 2). Region of Interest (ROI) annotation, segmentation, and classification labeling were performed by a physician with over 10 years of head and neck MRI experience on the EasyDL platform. This process involved: 1. Cropping Images to 480 × 480 pixels centered on ROI; 2. Manual segmentation of TMJ MRI into four labels: TMJ disc, condyle, temporal bone joint surface (including the glenoid fossa, and articular tubercle), and other tissues; 3. Classification of segmented and original images into four categories: N-CMP, N-OMP, ADD-CMP, and ADD-OMP. After labeling, to mitigate dataset bias, a physician with 22 years of experience reviewed the images to resolve any discrepancies through discussion until a consensus was reached. The consensus images formed the dataset, and the sample size for four categories was standardized.

### 2.4 Model construction and deployment

Deep learning allows the network to autonomously select features without manual intervention, necessitating a substantial volume of data for model training. In this study, the deep learning algorithm was constructed using Python version 3.11.8 (Python Software Foundation, DE, United States). The implementation utilized the ResNet101_vd deep learning framework with EasyDL as the backend platform. The computational environment comprised a single NVIDIA Tesla P40 GPU (24 GB VRAM), a 12-core CPU, and 40 GB of RAM. ResNet101_vd, a deep residual network with 101 layers, was designed with various parameter combinations to enhance model performance. EasyDL, developed by Baidu, facilitated the complete process of model creation, data upload, model training, and deployment.

Four different deep-learning models were trained. The first model was an ROI detection model, centered on the condyle and including the TMJ disc, condyle, temporal bone joint surface, and other tissues, it was extracted and classified into four categories. The second model segmented the TMJ region using augmented ROI MR images. After segmentation on EasyDL, four labels were utilized: TMJ disc, condyle, articular tubercle and fossa, and other tissues. Data augmentation techniques included shear, translation, rotation, sharpening, color posterization, and brightness adjustment. The output files, saved in JSON format, were used for training the fourth model. The third and fourth models were ADD classification models. The third model is a non-segmentation classification model, directly identifying normal disc-condyle relationships and ADD using augmented ROI MR images. The fourth model is a segmentation-based classification model, using segmentation from the second model to identify four conditions: N-CMP, N-OMP, ADD-CMP, and ADD-OMP. Training parameters included a base learning rate of 3e-5, epochs of 150, evaluation interval of 10, batch size of 1, and the optimizer AdamWDL (Adam with Layer-wise Adaptive Learning Rates). After classification by the third and fourth models, diagnoses were categorized as normal, Anterior Disc Displacement with Reduction (ADDWR) and Anterior Disc Displacement without Reduction (ADDWoR), based on images captured with both the mouth open and closed. To aid in interpreting the diagnostic principles, an artificial intelligence technology interpretation model with visualized heatmaps was employed using the Grad-CAM (Gradient-weighted Class Activation Mapping) visualization scheme. CAM images are heatmaps generated from the input images and superimposed onto the original images to represent the importance of each pixel to a specific output class (Qian et al., 2023). Integration of the trained models and parameters was performed using Python and deployed via EasyDL, enabling the application of these models for clinical diagnosis of TMJ ADD.

### 2.5 Performance metrics

In this research, a comprehensive set of performance metrics was utilized to assess the efficacy of the deep learning models. This assessment included metrics like precision, recall, mean average precision (mAP), the F1-score, the Matthews Correlation Coefficient (MCC), and an examination of confusion matrices, ensuring a thorough quantification of model performance.

#### 2.5.1 Precision

This metric reflects the proportion of accurately identified true positive cases out of all predicted positive instances, and is calculated as follows:

$$\text{Precision}=\frac{\text{True}\;\text{Positives}\;}{\text{True}\;\text{Positives}\;+\text{False}\;\text{Positives}}$$

#### 2.5.2 Recall

Quantifies the fraction of true positive cases accurately identified by the model among all actual positive instances. It is calculated using the formula:

$$\text{Recall}=\frac{\text{True}\;\text{Positives}}{\text{True}\;\text{Positives}+\text{False}\;\text{Negatives}}$$

#### 2.5.3 Mean Average Precision (mAP)

Defined as the arithmetic mean of the Average Precision (AP) scores across all classes, mAP synthesizes the overall performance of a classification model. AP is computed from the area under the precision-recall (P-R) curve, which is created by adjusting the decision threshold. The mAP formula is presented here:

$$mAP=\frac{1}{N}\sum_{i=1}^{N}{AP}_{i}$$

where N represents the total number of classes, and APi represents the average precision for the *i*th class. The mAP spans a scale from 0 to 1, with values closer to 1 indicating superior model performance across all classes.

#### 2.5.4 F1-score

The F1-score provides a balanced measure by combining precision and recall through their harmonic mean, capturing the balance between false positives and false negatives. It is calculated as:

$$F1=2\times \frac{\text{Precision}\times \text{Recall}}{\text{Precision}+\text{Recal}}$$

#### 2.5.5 MCC

A value that ranges from −1 to 1, used to measure the performance of binary or multiclass classification models, providing a balanced metric that reflects both the model’s sensitivity (True Positive Rate, TPR) and specificity (True Negative Rate, TNR). The formula for calculating MCC is:

$$\text{MCC}=\frac{\left(TP\times TN\right)-\left(FP\times FN\right)}{\sqrt{\left(TP+FP\right)\left(TP+FN\right)\left(TN+FP\right)\left(TN+FN\right)}}$$

MCC takes into account the true positives (TP), false positives (FP), true negatives (TN), and false negatives (FN) of the model’s predictions, a higher MCC value indicates better model performance. An MCC of 0 indicates that the model’s performance is equivalent to random guessing, while an MCC of 1 indicates perfect model predictions.

#### 2.5.6 Top-1 accuracy

These metrics are instrumental in assessing the efficacy of multi-class classification models. Top-1 accuracy indicates the proportion of instances where the model’s highest probability prediction corresponds to the correct class.

These metrics collectively furnish a robust assessment of model performance, enabling a nuanced understanding of the models’ diagnostic capabilities across various dimensions.

## 3 Results

**FIGURE 3:**
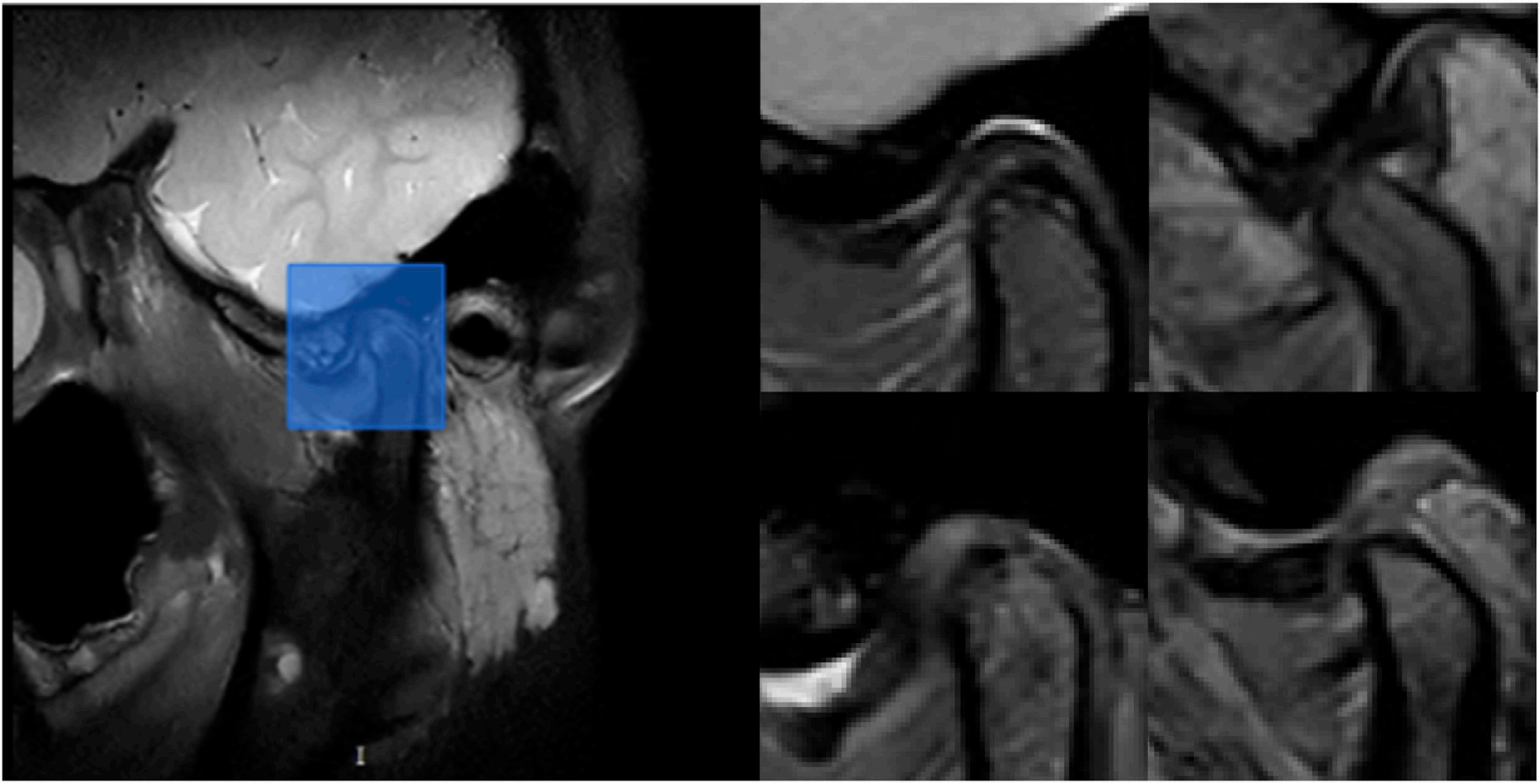
Example of ROI detection. On the left, the blue rectangle indicates the retained region after model prediction. On the right, the representative images show the retained regions after model prediction for the following conditions: N-CMP, N-OMP, ADD-CMP, and ADD-OMP, respectively.

**FIGURE 4:**
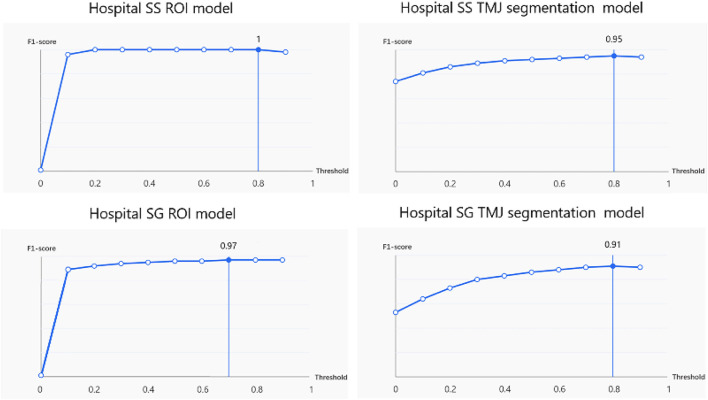
F1-score under different thresholds for the ROI model and TMJ segmentation model.

The ROI detection model consistently identified a minimal region sufficient for diagnosing TMJ ADD (Figure 3), including regions retained after predictions for N-CMP, N-OMP, ADD-CMP, and ADD-OMP. In the internal validity test conducted at Hospital SS, the model achieved a mAP, precision, and recall of 100%, with an F1-score of 1 at a threshold of 0.8. In the external validity test conducted at Hospital SG, the model’s performance was slightly lower but still achieved good results. The model achieved a mAP of 97%, precision of 95.8%, recall of 97.9%, and an F1-score of 0.97 at a threshold of 0.7 (Figure 4).

**FIGURE 5:**
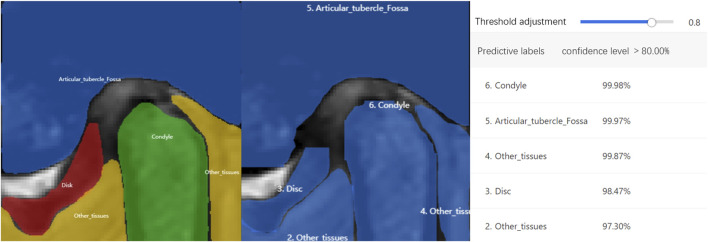
Representative examples of segmentation models.

**FIGURE 6:**
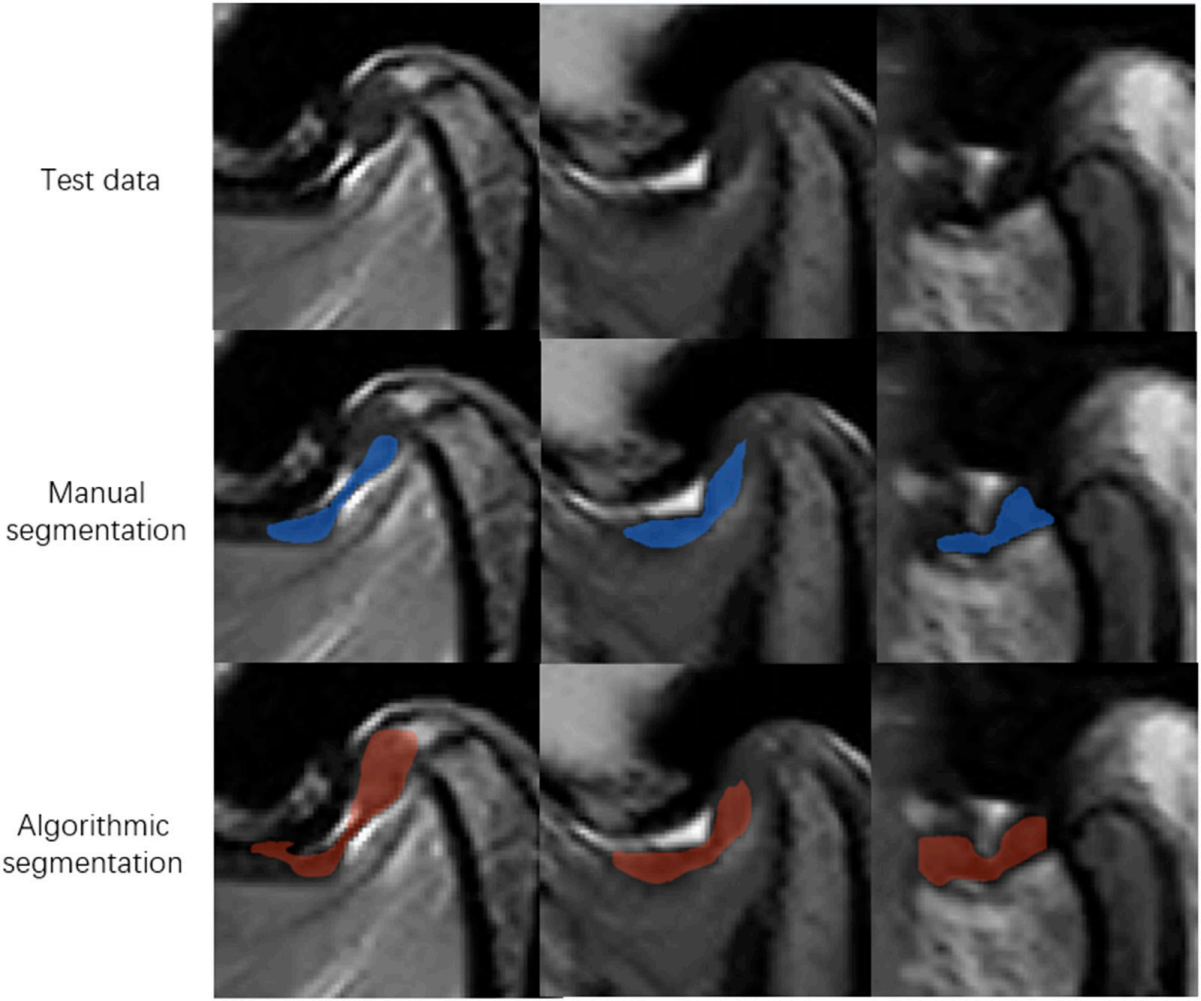
Representative images of model missegmentation. The first row shows the original MR images. The second row presents the manually segmented images by experts (blue regions). The third row depicts the images with model missegmentation (red regions).

At Hospital SS, the internal validity test demonstrated that the TMJ segmentation model had a mean Average Precision (mAP) of 94.86%, a precision of 94.05%, a recall of 95.11%, and an F1-score of 0.95 at a threshold of 0.8. For the external validity test conducted at Hospital SG, the model’s performance was slightly lower but still resulted in satisfactory outcomes. The model achieved a mAP of 90.4%, a precision of 91.4%, a recall of 90.7%, and an F1-score of 0.91 at the same threshold of 0.8. The average recognition rates for the TMJ disc, condyle, articular tubercle fossa, and other tissues were 83.43%, 100%, 96.01%, and 100%, respectively. Representative examples of segmentation are shown in Figure 5, demonstrating close alignment between model predictions and manual segmentations. We assessed the algorithm’s performance, finding higher accuracy in segmenting hard tissues such as the glenoid fossa and articular tubercle, with the disc being the most challenging to identify (Figure 6).

Based on the criteria for ADD, we included additional confusing images for incremental training to enhance the model’s ability to differentiate borderline cases for the two classification models. Both Model 3 and Model 4 exhibited high performance. However, to enhance overall diagnostic accuracy, when evaluating the combined results from both internal validity tests at Hospital SS and external validity tests at Hospital SG, the segmentation-based classification model significantly improved the predictive accuracy, as shown in Table 1, the overall predictive accuracy further increased from 0.9220 to 0.9691.

**TABLE 1:** Performance Metrics of two classification models.

Model	Hospital	Top1 accuracy	Precision	Recall	F1-score
Non-segmentation classification model	Hospital SS	95.82%	95.86%	95.96%	95.85%
	Hospital SG	88.58%	88.57%	88.67%	88.59%
	Total	92.20%	92.22%	92.32%	92.22%
Segmentation-based classification model	Hospital SS	99.53%	99.57%	99.58%	99.57%
	Hospital SG	94.29%	94.29%	94.38%	94.30%
	Total	96.91%	96.93%	96.98%	96.94%

Additionally, there was a noticeable improvement in the precision and F1 score for each classification category (Table 2). The MCC for the binary classifications of N-CMP versus ADD-CMP and N-OMP versus ADD-OMP is presented in Table 3. In the classification task of TMJADD, the models from Hospital SS demonstrated outstanding performance at Hospital SS, with MCC values ranging from 0.939 to 0.953 for differentiating N-CMP from ADD-CMP and 1.000 to 0.942 for distinguishing N-OMP from ADD-OMP. This indicates a high level of discriminative ability and generalizability. In contrast, the models from Hospital SG exhibited slightly lower MCC values of 0.795 to 0.853 and 0.858 to 0.942 for the same classification tasks, yet still demonstrated robust distinguishing and generalization capabilities. These results confirm the overall reliability of the model in providing effective diagnostic support for clinical practice.

**TABLE 2:** Performance Metrics for each classification of two classification models.

	Classification	Hospital	Precision	F1-score	Recall
Non-segmentation classification model	N-CMP	Hospital SS	100.00%	96.15%	92.59%
		Hospital SG	85.71%	88.24%	90.91%
		Total	92.86%	92.19%	91.75%
	N-OMP	Hospital SS	97.14%	96.87%	96.59%
		Hospital SG	91.43%	91.43%	91.43%
		Total	94.29%	94.15%	94.01%
	ADD-CMP	Hospital SS	93.71%	94.80%	95.91%
		Hospital SG	88.57%	86.11%	83.78%
		Total	91.14%	90.45%	89.85%
	ADD-OMP	Hospital SS	92.57%	95.58%	98.78%
		Hospital SG	88.57%	88.57%	88.57%
		Total	90.57%	92.07%	93.68%
Segmentation-based classification model	N-CMP	Hospital SS	100.00%	100.00%	100.00%
		Hospital SG	91.43%	92.75%	94.12%
		Total	95.71%	96.38%	97.06%
	N-OMP	Hospital SS	100.00%	100.00%	100.00%
		Hospital SG	97.14%	97.14%	97.14%
		Total	98.57%	98.57%	98.57%
	ADD-CMP	Hospital SS	100.00%	99.15%	98.31%
		Hospital SG	94.29%	91.67%	89.19%
		Total	97.14%	95.41%	93.75%
	ADD-OMP	Hospital SS	98.29%	99.14%	100.00%
		Hospital SG	94.29%	95.65%	97.06%
		Total	96.29%	97.39%	98.53%

**TABLE 3:** MCC for N-CMP vs. ADD-CMP binary classification and N-OMP vs. ADD-OMP binary classification models.

		Hospital	True-positive	False-positive	False-negative	True-negative	MCC
non-segmentation classification model	N-CMP VS ADD-CMP	Hospital SS 700	175	0	11	164	0.939
		Hospital SG 140	30	4	3	31	0.795
	N-OMP VS ADD-OMP	Hospital SS 700	170	2	6	162	0.953
		Hospital SG 140	32	2	3	31	0.853
segmentation-based classification model	N-CMP VS ADD-CMP	Hospital SS 700	175	0	0	175	1.000
		Hospital SG 140	32	3	2	33	0.858
	N-OMP VS ADD-OMP	Hospital SS 700	175	0	0	172	1.000
		Hospital SG 140	34	1	1	33	0.942

**FIGURE 7:**
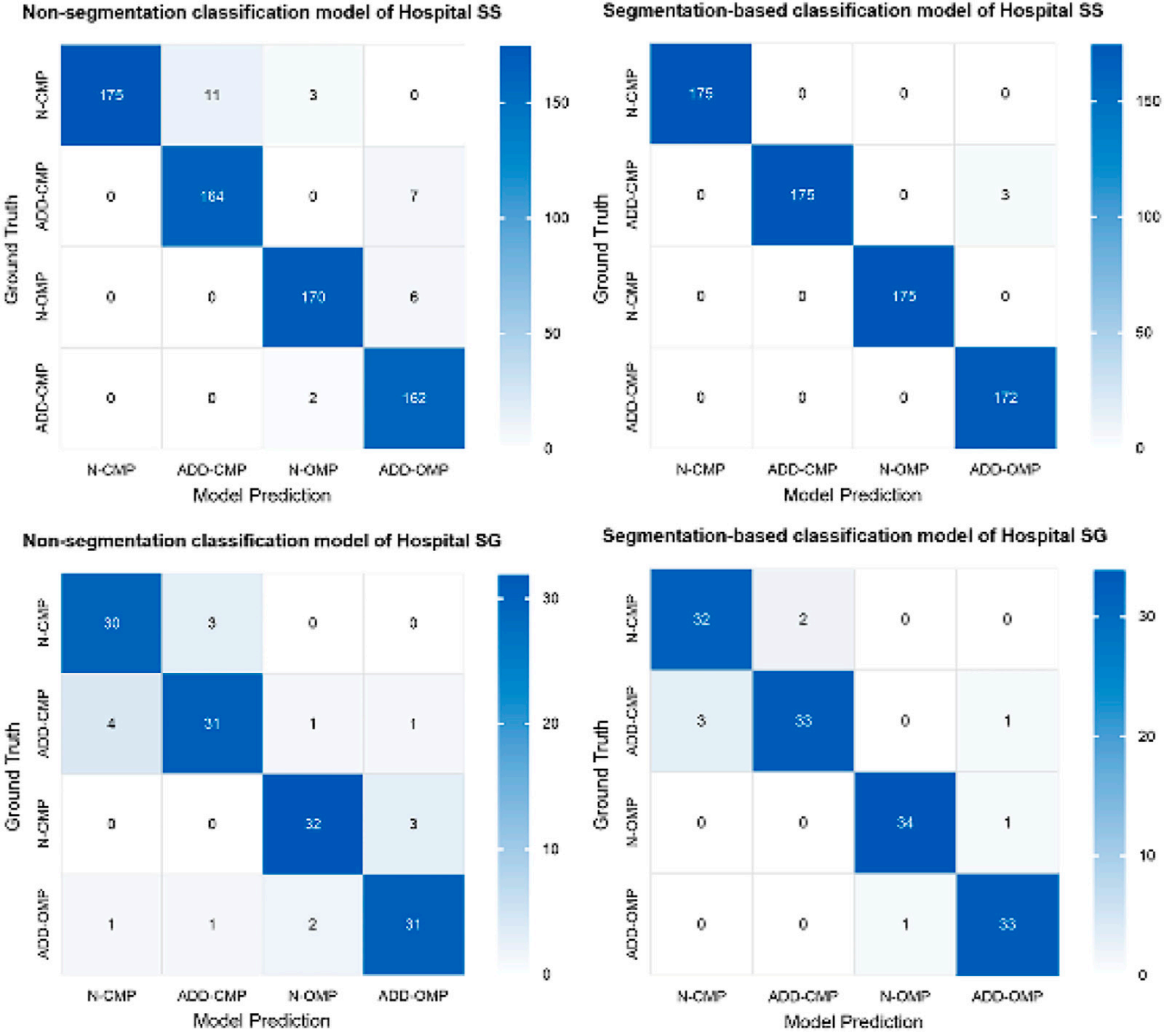
Confusion matrix heatmaps for Models 3 and 4.

**FIGURE 8:**
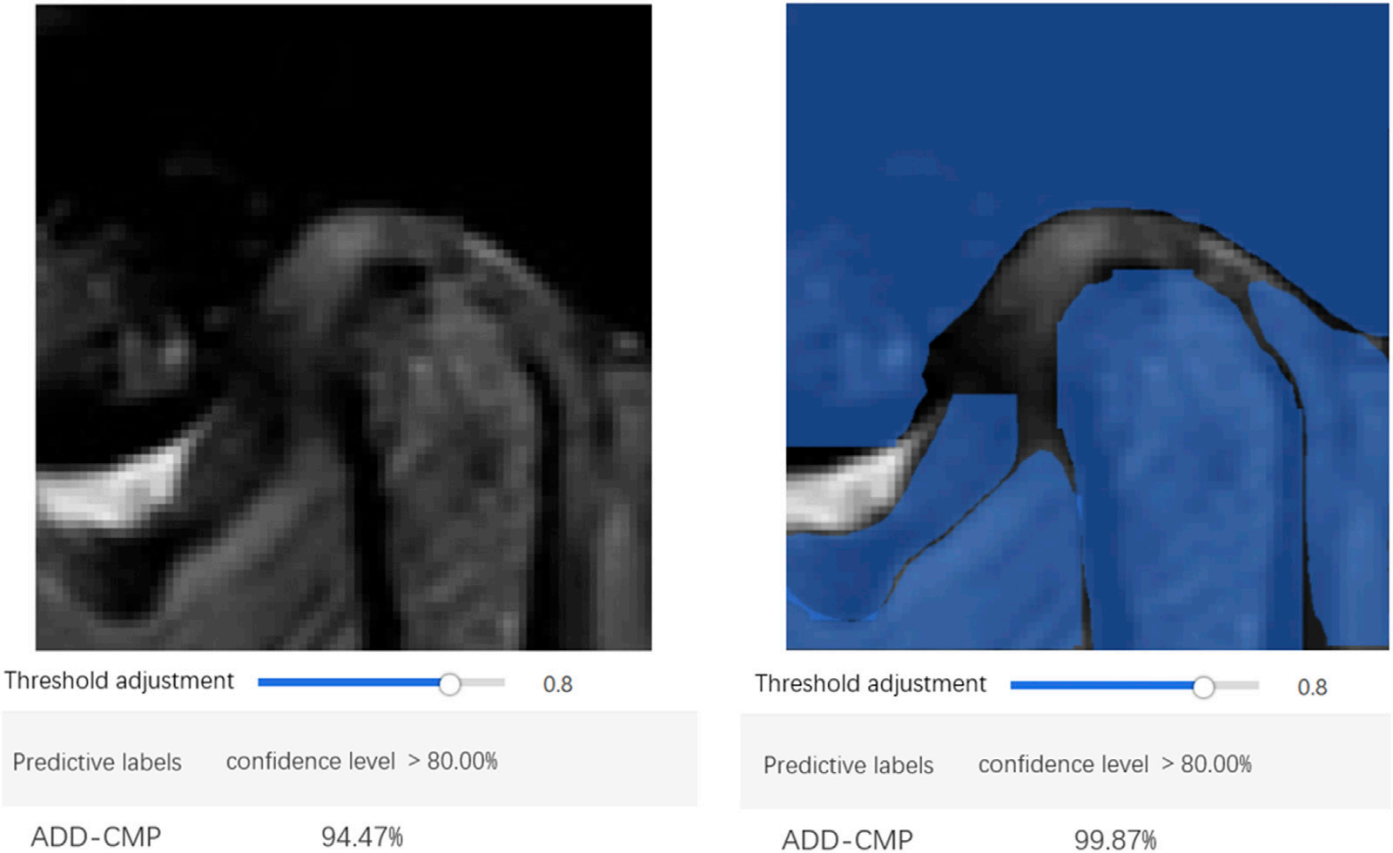
Representative image examples that typify the performance of Models 3 and 4.

The confusion matrix heatmaps for Model 3 and Model 4 are illustrated in Figure 7. Representative images for these models are provided in Figure 8.

EasyDL has developed a mobile application allowing users to capture TMJ MRI images using a smartphone camera or upload MRI images from a computer. The application leverages the trained models to perform real-time image recognition, providing image classification and confidence scores. Diagnosis based on the integration of MR images from both the open-mouth and closed-mouth positions can ascertain the condition as normal, ADDWR, or ADDWoR.

## 4 Discussion

Despite the capability of MRI to provide definitive diagnoses for TMJ disc disorders, previous studies have indicated poor reproducibility in TMJ MRI diagnostics due to variations in physician experience (Coombs et al., 2019; Costa et al., 2008). This inconsistency arises from the fact that ADD is often accompanied by disc deformation, perforation, and fibrosis, which obscure the disc boundaries on MRI. The morphology and positioning of the disc exhibit substantial variability among patients, complicating the accurate detection and segmentation of the disc.

Over the past decade, the utilization of deep learning techniques within the medical field has become increasingly widespread. Deep learning consists of computational models with multiple processing layers. For instance, in this study, Model 2 is a neural network trained to identify structures in the TMJ region, such as the articular disc. Each image, sized 480 × 480 pixels (totaling 230,400 pixels), is fed into the input layer, which consists of 230,400 neurons corresponding to each pixel. The output layer consists of neurons equal in number to the labels. Between the input and output layers, one or more hidden layers are present to process the data. Each neuron’s input in a given layer is the output from one or more neurons in the previous layer. Due to the multiple layers, these models are termed deep neural networks. Information passes from one layer to another through channels, each with associated weights. Each input value is multiplied by its respective weight, summed, and added to a bias value. This outcome is subsequently processed by a nonlinear function known as the activation function, which determines the activation state of the neuron. Neurons that are activated relay information to the subsequent layer. This process continues until the penultimate layer, where the activated neurons in the output layer represent the content of the input image. During training, weights and biases are continually adjusted until the model achieves optimal performance, thus enabling the neural network to analyze MRI images and provide reproducible and accurate results.

Our study introduces several innovative aspects of the field: 1. For the first time, we integrated four deep learning algorithms for three-stage multitask learning in TMJ ADD MRI image recognition; 2. We compared and cross-validated our segmentation-based classification model with the non-segmentation classification model to enhance diagnostic accuracy; 3. For cross-checking, our study tested the model on completely independent datasets from two hospitals; 4. We applied the industrial EasyDL platform to the field of TMJ MRI examination, deploying the model on mobile devices, thereby facilitating convenient TMJ ADD classification diagnosis through smartphone image capture or upload in clinical settings.

Our findings indicate that for the complex anatomical region of the TMJ in MRI, segmentation models can deeply understand the image content by extracting detailed information and eliminating background noise. Utilizing the results of image segmentation models for subsequent image classification enhances both the accuracy and robustness of the classification process. However, these advantages come with certain trade-offs, including increased computational costs, the requirement for larger training datasets, and extensive annotation.

In analyzing classification model errors, we found that borderline cases and restricted mouth opening, which blurs the distinction between open and closed-mouth images, were the primary causes of low recognition rates. This suggests that incorporating geometric judgment based on a coordinate system according to diagnostic standards may improve accuracy in identifying borderline cases in future studies. The recall rates of all models were lower when tested with images from Hospital SG compared to Hospital SS. This discrepancy may be due to the models being exclusively trained on data from Hospital SS, resulting in diminished external validity. Variations in imaging features across data domains could contribute to this issue. Model performance can be influenced by several factors. For instance, MRI scanners from different hospitals vary in magnetic field strength, resolution, and contrast, affecting image quality and the accuracy of feature extraction. Additionally, variations in imaging protocols, including slice thickness and acquisition angles, as well as environmental conditions during MRI scanning, operator expertise, and post-processing methods, can impact image quality. Demographic differences in patient populations across hospitals, such as age, gender, and disease severity, may also affect image presentation. These factors underline the necessity of ensuring dataset diversity and quality for enhancing model generalizability in medical image analysis across different hospitals. Future research should integrate data from multiple hospitals for model training and utilize data augmentation techniques to simulate diverse imaging conditions. This approach will enhance the model’s adaptability to varied data and improve its accuracy and reliability in clinical settings. Furthermore, fine-tuning models based on specific application requirements is essential for optimizing performance.

Despite the observed discrepancies, the recall rates at both hospitals exceeded 88%, indicating the models’ potential for clinical applicability and their high external validity. The accuracy of both hospitals exceeded 85%, however, the positive predictive value was lower for data from Hospital SG compared to Hospital SS. This discrepancy can be attributed to a higher incidence of false positives recorded in SG Hospital, particularly in the bilaminar zone in the closed-mouth position and the articular tubercle region in the open-mouth position. The connection between the posterior band of the articular disc and the bilaminar zone may contribute to this phenomenon, as the normal bilaminar zone consists of loose connective tissue that appears hyperintense signal. Anterior displacement of the disc is typically associated with morphological abnormalities, as well as a thickening in the retrodiscal area of the TMJ. Disease progression may lead to disc rupture, resulting in bone-to-bone contact and ultimately causing blurred posterior band boundaries of the TMJ disc on MRI. Additionally, the characteristics of PDWI may also influence the outcomes, indicating that future studies should consider integrating various imaging sequences for model training.

Previous studies have highlighted the limitation of deep learning models in that they provide prediction results without logically explaining the derivation process (Ozsari et al., 2023). Grad-CAM heatmaps address this by illustrating the importance of pixels influencing model decisions. The color map in Figure 1 shows that colors closer to the right indicate higher pixel importance. Grad-CAM maps for Models 3 and 4 reveal that the areas relied upon for classification are the disc and condyle regions, suggesting that the model classifies MRI by analyzing the position of the disc in relation to adjacent structures, such as the condyle and the articular tubercle. This recognition pattern parallels the diagnostic approach employed by clinicians to classify TMJ ADD, indicating that the model achieves the anticipated performance standards.

Given the critical importance of accurate TMJ ADD diagnosis, a deep learning-based TMJ ADD recognition system will provide convenience for clinicians, enhancing diagnostic and treatment accuracy and effectiveness for young physicians and primary healthcare facilities.

Although the developed models demonstrate high accuracy, our study has several limitations: 1. Two-Center Data and Limited Dataset: The dataset was sourced from two centers, and the limited number of images used for training may not adequately reflect the variations in hardware and imaging techniques across different medical institutions. This limitation could hinder the model’s generalizability and introduce bias. Furthermore, the model may not fully capture the diversity of TMJ conditions across different populations or healthcare settings. To address these limitations, future research should incorporate multi-center datasets and employ transfer learning and federated learning approaches to enhance the model’s generalizability and adaptability, ensuring it meets the needs of diverse populations and healthcare environments. 2. Limited to Sagittal MRI Images: The developed models are designed to evaluate sagittal MRI images. However, precise diagnosis of TMJ disc displacement frequently necessitates a thorough interpretation of images from both sagittal and coronal planes. Consequently, the current models are not optimized for identifying less common posterior and mediolateral displacements that may be clinically relevant; 3. Opaque Data Processing with EasyDL: The data processing workflow using EasyDL and the application functions as a “black box,” preventing us from gaining detailed insights into the processing steps.

To enhance and validate model performance, further development of deep learning algorithms is necessary for the comprehensive identification of multi-planar MR image data. Additionally, extensive multicenter studies are essential to enhance and confirm the model’s robustness and accuracy.

In summary, deep learning technologies, exemplified by Convolutional Neural Networks, have demonstrated substantial promise in multiple domains. We believe these advancements will play an increasingly crucial role in diagnostic research and clinical practice of TMDs.

## Data Availability

The raw data supporting the conclusions of this article will be made available by the authors, without undue reservation.
